# Gene flow and demographic history of leopards (*Panthera pardus*) in the central Indian highlands

**DOI:** 10.1111/eva.12078

**Published:** 2013-06-06

**Authors:** Trishna Dutta, Sandeep Sharma, Jesús E Maldonado, Thomas C Wood, Hemendra S Panwar, John Seidensticker

**Affiliations:** 1 Smithsonian Conservation Biology Institute, National Zoological ParkWashington, DC, USA; 2 Department of Environmental Science and Policy, George Mason UniversityFairfax, VA, USA; 3 Peace Institute Charitable TrustDelhi, India

**Keywords:** effective population size, forest corridors, gene flow, India, leopards, metapopulation, noninvasive genetics

## Abstract

Gene flow is a critical ecological process that must be maintained in order to counteract the detrimental effects of genetic drift in subdivided populations, with conservation benefits ranging from promoting the persistence of small populations to spreading adaptive traits in changing environments. We evaluated historical and contemporary gene flow and effective population sizes of leopards in a landscape in central India using noninvasive sampling. Despite the dramatic changes in land-use patterns in this landscape through recent times, we did not detect any signs that the leopard populations have been through a genetic bottleneck, and they appear to have maintained migration–drift equilibrium. We found that historical levels of gene flow (mean *m*_h_ = 0.07) were significantly higher than contemporary levels (mean *m*_c_ = 0.03), and populations with large effective population sizes (Satpura and Kanha Tiger Reserves) are the larger exporters of migrants at both timescales. The greatest decline in historical versus contemporary gene flow is between pairs of reserves that are currently not connected by forest corridors (i.e., Melghat-Pench *m*_h_ − *m*_c_ = 0.063; and Kanha-Satpura *m*_h_ − *m*_c_ = 0.054). We attribute this reduction in gene flow to accelerated fragmentation and habitat alteration in the landscape over the past few centuries, and suggest protection of forest corridors to maintain gene flow in this landscape.

## Introduction

Habitat fragmentation creates isolated populations that are prone to reduced population viability and ultimately greater risk of extinction (Lacy 1997; Gaggiotti 2003; Keyghobadi 2007). Gene flow between insular populations can mitigate these effects (MacArthur and Wilson 1967; Brown and Kodric-Brown 1977; Couvet 2002; Chiucchi and Gibbs 2010) by counteracting the negative effects of genetic drift and inbreeding (Keller et al. 2001; Ebert et al. 2002) and thus plays a crucial role in the persistence of natural populations (Bohonak 1999; Lenormand 2002). Movement of individuals between populations increases both local abundance (Brown and Kodric-Brown 1977) and genetic diversity (Westemeier et al. 1998; Madsen et al. 1999). Although there are some studies that have shown the disruptive effects of gene flow between locally adapted populations, several other studies have found positive fitness effects associated with gene flow, such as increased survival, recruitment, and population growth rate (Hedrick 1995; Vila et al. 2003; Postma and van Noordwijk 2005; Adams et al. 2011), especially in large ranging, conservation dependent, threatened species.

Previous studies have reported on the effect of habitat fragmentation on migration rates and effective population (*N*_e_) (Gill 1978; Palstra et al. 2007). Effective population size is a central concept in evolutionary and conservation biology because it determines the strength of stochastic evolutionary processes relative to deterministic forces (Crow and Kimura 1970). The effective population (*N*_e_) is the number of individuals in an idealized population (exhibiting random mating, discrete generations, no mutation, no migration, no selection) that would experience the same magnitude of genetic drift and exhibit the same rate of inbreeding as the biological population under consideration (Wright 1931). *N*_e_ is typically smaller than *N* (the population census size) when sex ratio is skewed or there are differences in individual contribution to reproduction. In the absence of gene flow, the rate of loss of genetic diversity via genetic drift is predicted to be greater in populations with small *N*_e_. Along with *N*_e_, measures of gene flow among different habitat fragments will help to understand the functionality and probability of persistence of a metapopulation. Such information is important to the management of wild populations and has been previously used to make specific recommendations for management interventions (e.g., Miller and Waits 2003; Tallmon et al. 2004).

Highly vagile carnivores are often long-range dispersers and can be expected to exhibit rates of gene flow sufficiently high to limit the accumulation of genetic differences between subpopulations (Wayne and Koepfli 1996). The leopard (*Panthera pardus*) is a habitat generalist and has the widest geographical distribution of all the *Panthera* cats. In the Indian subcontinent, leopards are sympatric with tigers, but are more widely distributed than the latter, partly because of their ability to inhabit a variety of forested and degraded habitats (Athreya et al. 2011) and to survive by feeding on relatively small prey such as domestic dogs, goats, and pigs in the absence of large wild prey such as deer (Seidensticker et al. 1990; Edgaonkar and Chellam 2002). Subadult leopards start exploratory movements independent of their mother at about 13 months of age, and dispersal typically takes place at 15–19 months of age (Sunquist 1983; Seidensticker et al. 1990). Leopard home range sizes vary with habitat types, prey densities, and tiger densities, from 6 to 13 km^2^ in Chitwan, Nepal (Sunquist 1983; Seidensticker et al. 1990), to 17–25 km^2^ in Nagarahole, India (Karanth and Sunquist 2000) and 26 and 45 km^2^ in adult females and males, respectively, in Huai Kha Khaeng, Thailand (Simcharoen et al. 2008). Although precise dispersal distances for leopards have not been measured, the cats are known to travel long distances until they find suitable habitat patches not inhabited by larger competitors such as tigers (*Panthera tigris*) or by other same-sex adult leopards (Bailey 1993). For example, a leopardess captured in one location and released in another site traveled 90 km through a heavily human-dominated landscape in peninsular India (Athreya et al. 2007).

**Figure 1:**
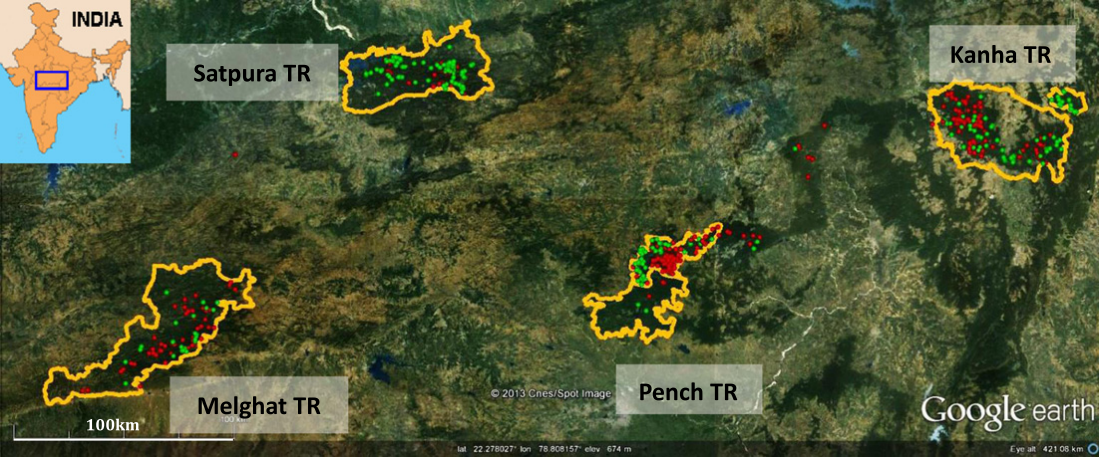
Map of the Satpura-Maikal landscape with its location in India (inset). Green dots represent location of individual leopards, and red dots represent individual tiger locations, in each Tiger Reserve (orange boundary) using multilocus genotype data. The inter-connecting corridors are also visible.

In developing countries such as India, which are undergoing rapid economic expansion and urbanization, the continued loss and fragmentation of forested habitat is virtually inevitable. As a result of anthropogenic activities, historically continuous habitats have been transformed into a mosaic of remnant forests embedded in an urban or agricultural matrix. The Satpura–Maikal landscape (Fig. 1) is one such mosaic of farmland, urban centers, and other forms of human-modified habitat interspersed with remnant forested areas that have been fragmented over the past several centuries. We studied a leopard metapopulation in this landscape, consisting of four populations present in five Tiger Reserves (TRs) that are interconnected by forested corridors. Using DNA extracted from field-collected fecal matter (scats), we derived temporal effective population sizes and the magnitude and directionality of gene flow in this metapopulation. Because leopards are habitat and prey generalists, we hypothesized that: (i) leopards in this landscape have high levels of gene flow, (ii) historical levels of gene flow were higher than contemporary gene flow, and (iii) historical population sizes were higher than current population sizes. Comparison of level of gene flow and effective population size over time can provide information on the direction and magnitude of the genetic processes that operate at a metapopulation level and the genetic functionality of existing forest corridors.

## Material and methods

### Sampling

The study landscape is located in the central Indian highlands and represents an area of approximately 45 000 km^2^ in the Satpura-Maikal landscape (21.15 N–76.50 E, 22.80 N- 81.05 E). During April–June 2009 and Nov 2009–May 2010, we conducted stratified random sampling in approximately 15 000 km of forest trails and roads, collecting felid (leopard and tiger) scats from four populations in five TRs, their buffer zones, and interconnecting corridors. The TRs are: Satpura (1428 km^2^, MP); Melghat (1677 km^2^, Mh), Pench (758 km^2^, MP/258 km^2^, Mh), and Kanha (2059 km^2^, MP). We put more effort in the TRs than in buffer and corridor areas so as to maximize the number of samples from different individual leopards. We identified felid scats by their morphology and associated signs such as scrapes and pugmarks. The GPS location of each scat was recorded. Satpura and Melghat are located to the west of this landscape, and Kanha and Pench are located to the east, and these two pairs of TRs are connected by a forest corridor each (Fig. 1). Satpura is also connected by a fragmented corridor to Pench. The presence of leopards and their prey species has been reported in these corridors (Jhala et al. 2011). The intervening matrix of the eastern and western pairs of TRs is dominated by farmland, urban centers, and other areas of high human density.

### Genetic methods

We extracted genomic DNA from scats using the QIAamp mini-stool kit (Qiagen Inc., Valencia, CA, USA) and used species-specific mitochondrial primers (Mondol et al. 2009) to distinguish leopard scats from those of tigers. We used a panel of highly polymorphic microsatellites developed from domestic cats and tigers to identify individuals (Table ST1; Dutta et al. 2012a) from leopard-positive samples. We used sterile conditions and precautions to reduce contamination and used the modified multitube approach (Taberlet et al. 1996) to account for scoring and amplifying errors. Details of genotyping, success, and error rates are available in Dutta et al. (2012a).

### Data analysis

We measured genetic diversity by estimating the number of alleles per locus (A), observed (Ho), and expected (He) heterozygosity in CERVUS 2.0 (Marshall et al. 1998). We conducted tests for deviations from Hardy–Weinberg equilibrium (HWE) and linkage disequilibrium (LD) using GENEPOP 4.0.10 (Rousset 2008) with a Bonferroni correction (Rice 1989) applied for multiple comparisons. We also used GENEPOP to calculate the effective number of migrants Barton and Slatkin (1986) between each pair of populations at a contemporary timescale. We used ARLEQUIN with 10 000 permutations to test the statistical significance of pairwise *F*_ST_ values (Weir and Cockerham 1984) as a measure of genetic differentiation among the four TRs.

We used BAYESASS1.3 (Wilson and Rannala 2003) and MIGRATE (Beerli and Felsenstein 2001; Beerli 2006), respectively, to estimate contemporary (past few generations) and historical gene flow (much longer period of time, approximately 4 *N*_e_ generations in the past; Beerli and Felsenstein 2001). We take contemporary timescale to be about five generations, or 20–25 years (assuming a generation time of 4–5 years for leopards) and the historical timescale to be several hundreds of years before present (YBP).

BAYESASS uses a Bayesian method with Markov Chain Monte Carlo (MCMC) to measure gene flow by identifying population-specific inbreeding coefficients and genotypic disequilibrium. It assumes linkage equilibrium and that populations have not been subjected to genetic drift within the past 2–3 generations before sampling and allows deviations from Hardy–Weinberg expectations within populations. We used 3 × 10^6^ iterations, with a burn-in of 10^6^ iterations, and a sampling frequency of 2000 to ensure that the model's starting parameters were sufficiently randomized (confirmed by checking changes in likelihood values). Delta values were adjusted to optimize terminal proposed changes between chains (40%–60% of the total iterations) to ensure sufficient parameter space was searched (Wilson and Rannala 2003). We performed five runs using different starting-seed values to ensure consistency between runs. BAYESASS provides mean and 95% confidence intervals (CI) expected for uninformative data that can be used to assess the reliability of data and also identifies first- and second-generation migrants in a population. We used ML-Relate (Kalinowski et al. 2006) to establish the relatedness between second-generation migrants.

MIGRATE uses coalescent theory to jointly estimate mutation-scaled migration rate [(*M*), *M* = *m*_h_/μ, where *m*_h_ is historical migration rate, μ is mutation rate per generation [10^−2^, (Driscoll et al. 2002)], and mutation-scaled effective population size [(Θ), Θ = *x*μ*N*_e_, where *x* is 4 for nuclear data and *N*_e_ is historical effective population size]. MIGRATE allows for unequal population sizes and asymmetrical migration, thereby more closely reflecting biological reality than traditional *F*-statistics-based methods (Palstra et al. 2007). It also allows deviations from Hardy–Weinberg expectations, but assumes that populations are in migration–drift equilibrium. We ran three replicates of MIGRATE using a Brownian motion mutation model with constant mutation rates and starting parameters based on *F*_ST_ calculations. We used slice sampling and uniform prior distribution to estimate Θ (range = 0–20, mean = 10) and *M* (range = 0–500, mean = 250, and delta = 250). Following a burn-in of 50 000 iterations, each run visited a total of 1 000 000 parameter values and recorded 20 000 genealogies at a sampling increment of 50. We used a static heating scheme at four temperatures (1, 1.5, 3, and 6) to efficiently search the genealogy space. In the results, we report the mean and 95% credible intervals for Θ and *M*. We also calculated the effective number of migrants using the relation (*N*_m_ = Θ × *M*/4) and added bidirectional values to be comparable to contemporary estimates.

We used the statistical approach developed by Ciofi et al. (1999) in the program 2-MOD, to test the likelihood of two models of population history: pure drift versus immigration–drift equilibrium. In the pure drift model, allele frequencies in each population are solely the product of drift, and the effect of migration between populations is negligible. Conversely, in the immigration–drift equilibrium model, allele frequencies within populations are determined by a balance between drift and immigration. 2-MOD uses an MCMC procedure to compare likelihoods of the two scenarios and produce probabilities of the data fitting each model. The MCMC simulation was run for 100 000 iterations, and we discarded the initial 10% of data to avoid biases introduced by the starting conditions. This program was run thrice to confirm the results.

We estimated contemporary effective population size using the programs LDNe1.31 (Waples and Do 2008) and ONESAMP1.1 (Tallmon et al. 2008), both of which require data from a single sampling session, unlike traditional *N*_e_ estimators that require temporally spaced genetic samples (Wang and Whitlock 2003). LDNe uses linkage disequilibrium (LD) information among alleles at different loci caused by genetic drift in populations. The linkage disequilibrium (LD) method is based on the expectation that LD will increase due to genetic drift generating nonrandom associations among unlinked loci more substantially in small compared with large populations (Hill 1981).This method does not assume random mating and corrects for biases associated with small sample sizes (England et al. 2006; Waples and Do 2008). We estimated *N*_e_ for varying levels of inclusion of rare alleles (*P*_crit_ values 0.05, 0.02, and 0.01) to compare consistency across results, but report estimates from alleles with a frequency ≥0.02 because this criterion provides a good balance between maximizing precision and minimizing bias with highly polymorphic loci like microsatellites (Waples and Do 2008). ONeSAMP calculates eight summary statistics and uses approximate Bayesian computation (ABC) to estimate *N*_e_ from a single population sample. We used different priors for *N*_e_ (2–50, 2–100, and 2–200) to verify that the results were robust to these changes.

In order to quantify habitat loss and fragmentation at a very coarse scale, we used the Anthrome 2.0 dataset that maps and characterizes global anthropogenic transformation of the terrestrial biosphere from 1700 to 2000 (Ellis et al. 2010). The global patterns of these anthropogenic transformations were classified into nineteen classes using *a priori* anthrome classification algorithm. We used ArcGIS 9.2 (Redlands, CA, USA) to delineate our study landscape from the global Anthrome 2.0 dataset and calculated the area under the consolidated three major land-cover/land-use classes (dense settlement, villages and croplands, and semi-natural wildlands) that are relevant to this study (1700, 1800, 1900, 2000 CE).

To see if we could detect signatures of a population bottleneck, we used the program BOTTLENECK (Piry et al. 1999), which tests for heterozygote excess as compared with that expected under mutation–drift equilibrium. First, we used the qualitative approach of allele frequency distribution test. If any deviation is observed from the normal *L*-shaped allele frequency distribution that normally arises in a population, a bottleneck may be suspected. We then used a quantitative approach based on the principle that allelic diversity in a population reduces faster than heterozygosity after going through a bottleneck, resulting in a relative excess of heterozygotes (Cornuet and Luikart 1996; Spencer et al. 2000). Significance of observed deviations was determined by a two-tailed Wilcoxon signed-rank test (Luikart and Cornuet 1998), under the two-phase mutation (TPM) model (with 30% of SMM) because it has been suggested to be the most suitable for microsatellites. We also used *M*-ratios to detect any genetic bottleneck in our data. *M*-ratio is the ratio of *k/r*, with *k* representing the number of alleles and *r* representing the allelic size range. As rare alleles are lost, *k* is reduced faster than *r*, and therefore, a low *M-*ratio relative to a critical value indicates population declines. We calculated the population-specific *M*-ratio with the software *M_P_val* (Garza and Williamson 2001). We compared this empirical value of *M*-ratio to the commonly used bottleneck threshold (0.68; Garza and Williamson 2001) as well as a simulated equilibrium distribution based on the two-phase model of microsatellite mutation. This simulated critical value (*M*_c_) was calculated by simulating 10 000 replicates in *critical_M* (Garza and Williamson 2001) using the mean size of nonstepwise mutations (Δ_g_) = 3.5, and the proportion of stepwise mutations (*p*_s_) = 90% as recommended by the authors.

Because both BOTTLENECK and *M*-ratio detect only recent and severe population declines (Girod et al. 2011; Peery et al. 2012), we also used MSVAR v 1.3 (Storz and Beaumont 2002), which detects long-term changes in population sizes using an MCMC-based simulation approach of the mutation-coalescent history to present-day genotypes by characterizing the posterior distribution of the parameters *N*_0_ (current population size), *N*_1_ (ancestral populations size), μ (mutation rate of all loci), and *t* (time since change in population size). We used different and wide priors for each locus for *N*_0_, *N*_1_, μ, and *t*. Each run was 2 × 10^9^ steps, with a burn-in of 10 000 steps and output every 10 000 steps. We used Tracer v1.5 (Rambaut and Drummond 2007) to estimate posterior distributions and the highest posterior densities (HPDs) of current and ancestral population size.

## Results

We collected a total of 1411 felid scat samples and used species-specific mitochondrial primers and identified 463 tiger-positive (Sharma et al. 2013) and 287 leopard-positive samples (Fig. 1). We then used a panel of seven highly polymorphic microsatellites to identify 217 individual leopards (Dutta et al. 2012a). All loci were polymorphic, with 10–18 alleles across the four populations. All loci were in HWE in Melghat, while one locus in Satpura TR, and four loci each in Pench TR and Kanha TR were not in HWE (Dutta et al. 2012b). Six pairs of loci of 84 pairwise comparisons (four pairs in Pench and two in Satpura) were in significant linkage disequilibrium after Bonferroni corrections, and these pairs were not consistent across the populations. Overall, the mean number of alleles was 13.3, mean observed (Ho) and expected (He) heterozygosities were 0.74 and 0.84, respectively, and all TRs had similar and comparable levels of polymorphism (Dutta et al. 2012b). Overall average *F*_ST_ value for this landscape was low (0.041, SD 0.009), and all pairwise *F*_ST_ values were low (0.032–0.057), but significant.

**Figure 2:**
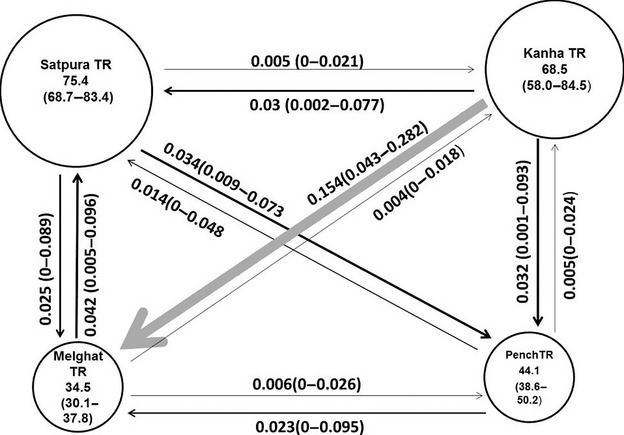
Contemporary gene flow (m, results from BAYESASS) and effective population sizes (*N*_e_, results from ONESAMP) in the central Indian leopard meta-population. Numbers inside circles represent effective population sizes, and those above arrows represent migration rates in the direction of the arrow. Numbers in brackets are 95% confidence intervals. Thickness of arrows and diameter of circles are scaled according to their values.

Gene flow estimation: Contemporary migration rates (*m*_c_) are highest from Kanha to Melghat (0.154) and lowest from Melghat to Pench (0.006) (Fig. 2). Because the estimated 95% confidence intervals in this analysis were much narrower than those expected for uninformative data (0.675–0.992), the estimates reported here can be assumed to be reliable. Except for the Kanha–Melghat pair, all other population pairs had overlapping 95% CI in both directions, indicating symmetric bidirectional gene flow. Gene flow from Kanha to Melghat is significantly higher than vice versa, and these migration rates seem to be at the higher end of our expectations because these two TRs are geographically the most distant from one another. Kanha has the highest net emigration rate (sum of outgoing gene flow minus the sum of incoming gene flow), even after excluding the disproportionately high migration rate from Kanha into Melghat. All the other TRs had negative net emigration rate (i.e., these TRs receive more migrants than they provide), with Melghat having the smallest net emigration value. Using assignment tests conducted in BAYESASS, we identified three first-generation migrants and six second-generation migrants. All first-generation migrants were in Satpura TR (two from Pench, and one from Melghat). One second-generation migrant was from Kanha to Satpura, and five second-generation migrants were from Kanha to Melghat. Two of the five migrant individuals from Kanha to Melghat were half-siblings, while the others were unrelated.

**Figure 3:**
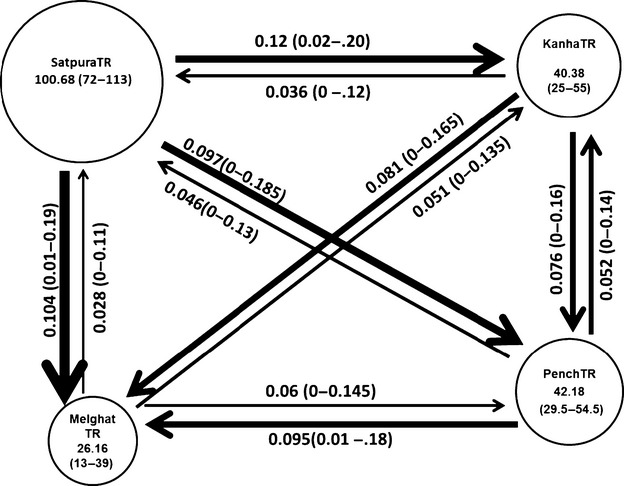
Estimates of historical gene flow(*m*) and effective population sizes (*N*_e_) in the four Tiger Reserves in the central Indian leopard meta-population (results from MIGRATE). Numbers inside circles represent effective population sizes, and those above arrows represent migration rates in the direction of the arrow. Numbers in brackets are 95% confidence intervals. Thickness of arrows and diameter of circles are scaled according to their values.

Mutation-scaled historical migration rates (*M*) ranged from 10.74 (Satpura to Kanha) to 2.75 (Melghat to Satpura) (Fig. 3). When we converted M into historical gene flow, mean *m*_h_ (0.07) was significantly higher than mean contemporary gene flow, *m*_c_ (0.03) (*P* = 0.008, one-tailed paired *t*-test), and the greatest decline between historical and contemporary gene flow is between Melghat-Pench (*m*_h_ − *m*_c_ = 0.063), followed by Kanha-Satpura (*m*_h_ − *m*_c_ = 0.054). Historical migration rates were highest from Satpura to Kanha (*M* = 10.74) and Satpura to Melghat (*M* = 10.36) and lowest from Melghat to Satpura (*M* = 2.75). No population pair had asymmetric bidirectional gene flow (nonoverlapping 95% CI, Fig. 3). Satpura had the highest emigration rates, and Melghat had the highest immigration rates. Analysis in 2-MOD revealed that all replicate runs selected the immigration–drift model [*P* (immigration–drift model) = 0.99]. *F* values, which represent the probability of alleles being identical by descent, were very low (0.01–0.07) in all the TRs (Table 1). Comparing the effective number of migrants provided additional support that migration rates have decreased in the contemporary timescale (Table ST2).

**Table 1:** Summary of sampling *F*-values and effective population sizes. *F* value (2 mod), *N*_e_ (Onesamp) are from priors of 2 to 100, *N*_e_ (LDNe) are from *P*_crit_ values of 0.2, Effective population size (Theta values) are from MIGRATE

Tiger Reserve	No. individuals	*F* value	Ne (Onesamp)	Ne(LDNe)	Theta(Migrate)	Ne(Historical)
Satpura	71	0.03	75.4 (68.7–83.4)	74.6 (50.4–125.8)	4.03 (2.88–4.52)	100.68
Melghat	35	0.01	34.5 (30.1–37.8)	86.4 (50.7–226.1)	1.05 (0.52–1.56	26.16
Pench	54	0.04	44.1 (38.8–50.2)	37.7 (24.3–66.5)	1.69 (1.18–2.18)	42.18
Kanha	57	0.07	68.5 (58.0–84.5)	73.7 (45.6–153.3)	1.62 (1–2.2)	40.38

Demographic history: Estimates of mutation-scaled effective population size Θ ranged from a minimum value of 1.05 in Melghat to a maximum of 4.03 in Satpura, whereas Kanha and Pench had similar values (1.62 and 1.69, respectively) (Table 1, Fig. 3). Estimates of contemporary *N*_e_ using the linkage disequilibrium and approximate Bayesian approaches were similar and comparable across TRs (Table 1). Satpura had the highest *N*_e_ using both approaches. Estimates for Melghat, using LDNe were more than twice the size of those obtained by ONESAMP. ONESAMP produced similar results for different priors, and here we report estimates from priors of 2 to 100. For LDNe, we report estimates from alleles with a frequency ≥0.02. In general, ONESAMP produced results with narrower 95% confidence limits than LDNe (Table 1).

The BOTTLENECK analysis revealed that the allele frequency distribution was normal (*L*-shaped), and there was no significant deviation in heterozygosity (*P* = 0.3) using the two-tailed Wilcoxon signed-rank test under the TPM model. The observed *M*-ratio value was always greater than the population-specific *M*_c_ (Table 2), as well as the widely used threshold of 0.68 (Garza and Williamson 2001). Analysis in MSVar showed no signs of significant population decline [*N*_0_ = 4.17 (95% HPD = 1.79–7.18), *N*_1_ = 4.26 (95% HPD = 1.78–5.35)], because 95% HPDs for both *N*_0_ and *N*_1_ were overlapping.

**Table 2:** The *M*-ratio (with theta = 10, proportion of larger mutations Δ_g_ = 3.5 and the proportion of stepwise mutations *p*_s_ = 90%) calculated for the different reserves in the meta-population. Observed *M* ratios are greater than the critical *M* (*M*_c_) in all populations

	Kanha	Melghat	Pench	Satpura
Observed *M*-ratio	0.856	0.847	0.858	0.889
Simulated critical *M* (*M*_c_)	0.693	0.665	0.689	0.700

Change detection analysis using the Ellis et al. (2010) data revealed major changes in the habitat over a period of 300 years (Table 3). We detected a 77% loss of forest cover, mainly to farmlands and urbanization. In the last 300 years, there has been a 21-fold increase in agricultural area and a 25-fold increase in urbanization, while human population has increased by 10 times in the same timeframe (Table 3).

**Table 3:** Changes in the characteristics of the studied landscape over four centuries (1700–2000)

Landscape characteristic	1700	1800	1900	2000
Dense settlement*	0	1	3	25
Villages & croplands*	37	266	486	805
Seminatural wildlands*	989	760	540	221
Human population†	100	160	255	1028

* Source Ellis et al. (2010). Numbers represent the number of cells in the study landscape belonging to each class.

† Source: Registrar General, India (2011).

## Discussion

The main findings of this study were that: (i) this metapopulation has been in migration–drift equilibrium; (ii) the population has not been through a severe demographic decline and shows no evidence of a recent genetic bottleneck; (iii) historical levels of gene flow were significantly higher than contemporary levels of gene flow; (iv) the largest decline in historical versus contemporary gene flow is between pairs of reserves that currently do not have adequate forest connectivity; (v) leopard populations with the largest effective population sizes are the largest exporters of migrants at both timescales; (vi) Melghat is a sink population at both timescales (it has the highest immigration rate); and (vii) historical and contemporary effective population sizes are similar in trend: Kanha and Satpura have larger estimates, and Melghat has the smallest estimates of population sizes at both timescales.

It is important to recognize that populations suffering a reduction in census size (demographic decline) may not necessarily suffer a severe reduction of *N*_e_ (a genetic bottleneck) and vice versa (Luikart et al. 1998). Bottlenecks generate detectable genetic footprints only in cases of extreme population decline, when effective population sizes undergo a 10–1000-fold decline, or populations are reduced to a very few individuals (*N*_e_ = 10–100) (Girod et al. 2011; Peery et al. 2012). Further, bottleneck detection can be obscured by multiple factors including prebottleneck genetic diversity, immigration, and timing and duration of the event, which relate to the generation time of the studied species (Cornuet and Luikart 1996; Garza and Williamson 2001; Williamson-Natesan 2005). Any cryptic change that we were not able to detect was probably not severe enough to produce a genetic bottleneck. Analysis in MSvar showed no signs that these leopard populations have undergone any significant demographic change. Given the dramatic changes in land-use patterns and fragmentation of habitat in this landscape (Table 3), we would expect to see the leopard population sizes decline over time. However, we should reiterate that MSvar would not have been able to detect a signature of demographic change unless the decline was rapid and rather severe (Girod et al. 2011). Thus, we caution against presuming that just because we did not find signatures of a genetic bottleneck in this leopard metapopulation, it does not imply that populations have remained stable, particularly if the decline was more gradual and persistent over time. We suggest conducting further analysis using high-resolution markers such as a large panel of SNPs using ancient DNA from museum specimens in order to better examine temporal changes of genetic diversity. We also recommend expanding the geographical area of sampling in order to provide a much more representative perspective of the species' genetic diversity.

Satpura had the largest value for Θ, and historically, it was the largest source of migrants. In the contemporary timescale, Kanha is the largest source of migrants, even when excluding the seemingly disproportionately high migration rate from Kanha into Melghat (Fig. 2). Melghat had the smallest Θ and contemporary *N*_e_, and highest immigration rates at both temporal scales (Figs 2 and 3). Thus, at both temporal scales, the populations with larger *N*_e_ are acting as the source populations; Melghat is a sink at both the temporal scales, while Kanha and Satpura are source populations at the contemporary and historical time frame, respectively. This source-sink effect is probably a reflection of topography and the history of land use in the landscape, combined with other forms of anthropogenic influence. India includes some of the oldest agrarian civilizations, and there are records of forests being cleared for cultivation even in 1300 BCE (Rangarajan 1999). All of these reserves lie in hilly regions of the central highlands, and historically, these locations may have served as refugia before they were declared protected areas. This argument is further supported by our change detection analysis, which shows major habitat change and loss of forest cover in this landscape over the past 300 years.

We addressed a range of assumptions and caveats of the analyses we used. One of the main assumptions of MIGRATE is that the populations have been in immigration–drift equilibrium and have had relatively constant population sizes (Beerli and Felsenstein 2001). Analysis in 2-MOD confirms that the four populations in this landscape have been in migration–drift equilibrium and that the current levels of genetic structure are a result of equilibrium between migration and drift. These leopard populations have not evolved in isolation due to genetic drift, and we did not detect any indication of this population having gone through any severe population decline leading to a genetic bottleneck. While current differentiation is low (Dutta et al. 2012b), it would be reasonable to expect this to increase if migration rates continue to decrease further.

We previously reported nine first-generation migrants in this landscape (Dutta et al. 2012b), using the migrant function in the programs STRUCTURE (Pritchard et al. 2000) and GENECLASS (Piry et al. 2004). However, analysis using BAYESASS identified three of these previously identified migrants as first-generation migrants; the other six were identified as second-generation migrants. One second-generation migrant was from Kanha to Satpura, five second-generation migrants were from Kanha to Melghat, and no first-generation migrants were found between these two pairs of reserves.

The contemporary levels of gene flow are higher from Kanha to Melghat than between any other pair of TRs, although these TRs do not have any direct forest connectivity and are geographically the farthest apart (370 km). We identified five second-generation migrants in Melghat from Kanha. Two of these individuals turned out to be half sibs. This result indicates that we have, in fact, sampled the progeny of four individuals from Kanha that had bred with Melghat leopards. Interestingly, the direction of gene flow is unidirectional, that is, from Kanha TR, which has higher effective population size.

We propose three possible explanations for the high gene flow pattern we observed from Kanha to Melghat. A few individuals may have successfully migrated from Kanha into Melghat in the recent past by the shortest direct route between the two parks. However, this seems a highly unlikely situation, given that the animal(s) would have to constantly pass through large tracts of high human density and activity, with little or no forest cover (Fig. 1). A more reasonable route would track forest cover from Kanha→Pench→Melghat. Because both Kanha and Pench are areas of high tiger density (Karanth et al. 2004), and leopards are present along the boundaries of these reserves (e.g., Pench in Fig. 1), leopards emigrating from Kanha may have been forced to venture beyond Pench and on to Melghat until they found suitable habitats to establish their territories. This seems to be a highly possible scenario, as previous studies have indeed shown that tigers are socially and behaviorally dominant over leopards (Karanth and Sunquist 2000), and leopard densities are inversely related to tiger densities (Steinmetz et al. 2013). The relatedness analysis and unidirectional gene flow from Kanha to Melghat lend support to this hypothesis. A third possibility could be ‘human-induced gene flow’, that is, caused by the translocation of leopards from or near Kanha to or near Melghat. Translocation of leopards that are potential or proven threats to humans and/or their livestock is a common practice in India (Athreya et al. 2011). It is important to point out that dispersal refers to the movement of an individual away from its natal site, and it does not necessarily result in gene flow, which requires successful reproduction in the receiving population. So while we did detect first-generation migrants in Pench from Kanha (Dutta et al. 2012b), we failed to detect any second-generation migrants in Pench. Instead, we have detected successful gene flow in the form of second-generation migrants from Kanha into Melghat.

Overall, the contemporary migration rates were almost half of the historical migration rates. The greatest decline in historical and contemporary gene flow is between Melghat and Pench (*m*_h_ − *m*_c_ = 0.063) followed by Kanha-Satpura (*m*_h_ − *m*_c_ = 0.054), the pairs of TRs between which the habitat is most highly fragmented (Fig. 1). Effective number of migrants has also decreased in the contemporary timescale (Table ST2), and although these two measures are not directly comparable, it does give us further support for the results of loss in contemporary gene flow. Discordance in gene flow estimates at the two temporal scales may be due to ecological reasons (i.e., differential reproductive contribution of migrants versus residents, as shown in Peery et al. 2010), or due to actual temporal variation in population dynamics, most likely due to habitat change (Austin et al. 2004).

We think that habitat fragmentation is most likely responsible for the observed patterns of gene flow. The central Indian highlands landscape has been severely modified over the past few centuries, leading to extreme fragmentation of historically contiguous habitat (Table 3, Rangarajan 1999). All of these habitat changes can explain the reduced contemporary gene flow in comparison with the estimated historical levels in this landscape. Leopard density has been reported to be negatively associated with unprotected areas and urban development (Balme et al. 2010; Gavashelishvili and Lukarevskiy 2008). Leopards dispersing outside of protected areas are also reported to be susceptible to high mortality rates due to natural and anthropogenic causes (Balme et al. 2010). Although leopards can move through somewhat disturbed areas, it is not their preferred habitat. They lurk around villages and human habitations, usually in the search for easy prey, because they are squeezed out of the best wild prey areas by their competitors.

Kanha and Satpura have consistently higher leopard population sizes than do Melghat and Pench. Except for Melghat, the TRs also have consistent contemporary population estimates in both of the approaches that were used. LDNe produced an estimate of effective population size of 86 individuals in Melghat with the widest 95% CI (Table 1) among all other estimates using both methods. ONESAMP, on the other hand, estimated 34 individuals and produced a narrow 95% CI. Several other studies have also reported that ONESAMP provides more reliable and precise results (Beebee 2009; Barker 2011; Phillipsen et al. 2011). Historically, Satpura had the highest effective population size, Kanha and Pench had intermediate values, and Melghat had the lowest estimate, similar to the results from the contemporary scenario. These values of Θ translated into historical effective population sizes that ranged from 26 (95% CI 13–39) in Melghat to 100 (95% CI 72–113) in Satpura, while Kanha (40, 95% CI 25–55) and Pench (42, (95% CI 30–55) had intermediate values. This analysis shows that the trends in population sizes have been similar across several hundred generations, but migration rates have significantly decreased.

## Conservation implications

Our study shows that this landscape is functional, and it supports a metapopulation of leopards, with dispersal and gene flow among the TRs, usually through forest corridors. There has been a significant reduction in contemporary gene flow in comparison with historical levels of gene flow. The discordance in historical and contemporary gene flow is more pronounced between reserves whose interconnecting matrix has been fragmented over the past few hundred years. Our study strongly suggests that habitat fragmentation can alter critical evolutionary and ecological processes that shape and maintain species persistence in a landscape. Although leopards are habitat and diet generalists, they are large carnivores that need adequate habitat and safe access to food. This landscape, while highly fragmented, still has some of the best forest habitat, prey densities, and forest connectivities between the different reserves in India. These corridors are occupied by prey and predators. To avoid many problems that may stem from declines in effective population sizes, the best option would be to sustain the ongoing natural immigration through protection and restoration of corridors. Maintaining and enhancing the connectivities between the populations would predictably maintain the effective population size and reduce the impacts of demographic and genetic stochasticity. However, with growing economic demands, anthropogenic activities, and development projects such as opening coal mines, widening of National Highway-7, which passes through the Kanha-Pench corridor threaten to sever corridors that interconnect and facilitate gene flow in this landscape. Our results show that although gene flow is low, several individuals are effectively dispersing, even over large distances. We recommend careful consideration of any proposed developmental activities in these corridors and suggest that these functional forest corridors be protected for the long-term survival of leopards and other species in this landscape.

## Data archiving statement

Dutta T, Sharma S, Maldonado J, Wood T, Panwar H, Seidensticker J (2013) Data from: Gene flow and demographic history of leopards (*Panthera pardus*) in the central Indian highlands. Dryad Digital Repository. doi:10.5061/dryad.16vm1.

## Supporting Information

**Table ST1.** Details of microsatellite markers used in the study.

**Table ST2.** Effective no. of migrants.
