# Supplementary material for: Gene flow and demographic history of leopards (Panthera pardus) in the central Indian highlands
Source: Evol Appl. 2013 Jun 6;6(6):949–59. doi: 10.1111/eva.12078 (PMC3779095; doi:10.1111/eva.12078)
Supplement: Supplementary file 1 [file eva0006-0949-SD1.pdf]

Table ST1: Details of microsatellite markers used in the study.

Locus	Reference	Species isolated	Chromosome no	Forward	Reverse
E7	Bhagavatula & Singh 2006	Tigers	- NA -	GCCCCAAAGCCCTAAAATAA	GCATGTCGGACAGTAAAGCA
FCA008	Menotti-Raymond et al 1999	Domestic cat	A1	ACTGTAAATTTCTGAGCTGGCC	TGACAGACTGTTCTGGGTATGG
FCA126	Menotti-Raymond et al 2000	Domestic cat	B1	GCCCCTGATACCCTGAATG	CTATCCTTGCTGGCTGAAGG
FCA279	Menotti-Raymond et al 2001	Domestic cat	C1	AGCCAAGTAATATTCCTCTGTG	GTCCATCCGCAGATGAATG
FCA304	Menotti-Raymond et al 2002	Domestic cat	A2	TCATTGGCTACCACAAAGTAGG	CTGCATGCCATTGGGTAAC
FCA672	Menotti-Raymond et al 2003	Domestic cat	F2	AAGTTGCTTGCACACACTGC	TCCAAGAGCCTTTTCAGTTAGG
F42	Menotti-Raymond et al 2004	Domestic cat	A1	CCCACGTGGCTAATCAAAT	CACTGCACAAATTAAGAGGC

References:

Bhagavatula J, Singh L (2006) Genotyping faecal samples of Bengal tiger *Panthera tigris tigris* for population estimation: a pilot
study. BMC Genet 7:48

Menotti-Raymond M, David VA, Lyons LA et al (1999) A genetic linkage map of microsatellites in the domestic cat (*Felis catus*). Genomics 57:9–23

Table ST2: Effective no. of migrants. Contemporary measures of Nem are above the diagonal (calculated in GENEPOP), and historic measures of Nem are below the diagonal (calculated from MIGRATE).

Effective no. migrants	Satpura	Melghat	Pench	Kanha
Satpura	--	2.6	5.3	3.1
Melghat	5.5	--	3.3	4.7
Pench	8.7	5.0	--	2.8
Kanha	7.9	4.2	5.3	--
